# Correction: Guest-responsive polaritons in a porous framework: chromophoric sponges in optical QED cavities

**DOI:** 10.1039/d0sc90163f

**Published:** 2020-08-05

**Authors:** Ritesh Haldar, Zhihua Fu, Reetu Joseph, David Herrero, Luis Martín-Gomis, Bryce S. Richards, Ian. A. Howard, Ángela Sastre-Santos, Christof Wöll

**Affiliations:** Karlsruhe Institute of Technology (KIT), Institute of Functional Interfaces (IFG) Hermann-von-Helmholtz Platz-1 Eggenstein-Leopoldshafen 76344 Germany ritesh.haldar@kit.edu christof.woell@kit.edu; Karlsruhe Institute of Technology (KIT), Institute of Microstructure Technology (IMT) Hermann-von-Helmholtz Platz-1 Eggenstein-Leopoldshafen 76344 Germany; Área de Química Orgánica, Instituto de Bioingeniería, Universidad Miguel Hernández Avda. de la Universidad, s/n, Elche 03202 Spain asastre@umh.es; Karlsruhe Institute of Technology (KIT), Light Technology Institute (LTI) Engesserstrasse 13 Karlsruhe 76131 Germany

## Abstract

Correction for ‘Guest-responsive polaritons in a porous framework: chromophoric sponges in optical QED cavities’ by Ritesh Haldar *et al.*, *Chem. Sci.*, 2020, DOI: 10.1039/d0sc02436h.

The authors regret that an acknowledgment was omitted from the published article. The omitted acknowledgement is given below.

Á. Sastre Santos thanks the Ministerio de Ciencia e Innovación of Spain and FEDER funds for the financial support (CTQ2017-87102-R).

The Royal Society of Chemistry apologises for these errors and any consequent inconvenience to authors and readers.

